# A visual code book--structured probability distributions in natural scenes

**DOI:** 10.1186/1471-2202-13-S1-P9

**Published:** 2012-07-16

**Authors:** Weibing Wan, Zhiyong Yong

**Affiliations:** 1 Brain and Behavior Discovery Institute, Georgia Health Sciences University, Augusta, Georgia, 30912, USA; 2 Department of Ophthalmology, Georgia Health Sciences University, Augusta, Georgia, 30912, USA; 3 Vision Discovery Institute, Georgia Health Sciences University, Augusta, Georgia, 30912, USA

## 

Natural visual scenes consist of objects of various physical properties that are arranged in three-dimensional (3D) space in a variety of ways. When projected onto the retina, visual scenes entail highly structured statistics, occurring over the full range of natural variations in the world. To deal efficiently with this full range of natural variations, the visual system may have to generate percepts according to the probability distributions (PDs) of visual variables underlying any stimulus [1-5]. What, then, are these PDs in natural scenes? In this work, we proposed that these PDs are the components of the grand joint PD of the physical world and the images on the retina (GPDWI). Thus, our approach is to decompose GPDWI into a large set of PDs without information reduction. We call these PDs a visual code book.

To examine this visual code book, we first sampled a large number of scene patches (~2 degrees of visual angle) from a database of high-resolution 3D natural scenes and fitted a concatenation of 8^th^ order polynomial functions to the 2D and 3D data in the patches. Using the fitted polynomial functions, we classified all the natural scene patches into a large set of 2D-3D natural scene structures that have distinctive distributions of ranges and/or luminance. Finally, we developed a PD for each of the 2D-3D natural scene structures. Since any joint 2D-3D natural scene patch is a combination of the samples of these PDs, they form a faithful representation of GPDWI and can be used a visual code book.

We used these PDs to estimate 3D scenes from 2D images and to categorize 2D-3D natural scenes. Our results showed that accurate 3D vision from a single monocular view is achievable in many situations and that near-human performance can be achieved on categorizing 2D-3D natural scenes. We thus conclude that the visual code book obtained here captures faithfully the extraordinarily complex 2D-3D natural scene statistics and supports a range of tasks of natural vision.
